# A joint effort over a period of time: factors affecting use of urate-lowering therapy for long-term treatment of gout

**DOI:** 10.1186/s12891-016-1117-5

**Published:** 2016-06-06

**Authors:** Jane C. Richardson, Jennifer Liddle, Christian D. Mallen, Edward Roddy, Samantha Hider, Suman Prinjha, Sue Ziebland

**Affiliations:** Research Institute for Primary Care and Health Sciences, Keele University, Keele, Staffs. ST5 5BG UK; Nuffield Department of Primary Care Health Sciences, University of Oxford, Gibson Building, 1st Floor, Radcliffe Observatory Quarter, Woodstock Road, Oxford, OX2 6GG UK

**Keywords:** Gout, Urate lowering therapy (ULT), Patient perceptions, Qualitative, Primary care

## Abstract

**Background:**

Although international guidelines encourage urate lowering therapy (ULT) for people who have more than two attacks of gout, only 30 % of patients are prescribed it and only 40 % of those adhere to the treatment. The aim was to explore reasons for this through an exploration of patient experience and understanding of ULT treatment for gout.

**Methods:**

A qualitative study was conducted throughout the United Kingdom. Narrative and semi-structured video-recorded interviews and thematic analysis were used.

**Results:**

Participants talked about their views and experiences of treatment, and the factors that affected their use of ULT. The analysis revealed five main themes: 1) knowledge and understanding of gout and its treatment; 2) resistance to taking medication; 3) uncertainty about when to start ULT; 4) experiences of using ULT; and 5) desire for information and monitoring.

**Conclusion:**

Patients’ understanding and experiences of gout and ULT are complex and it is important for clinicians to be aware of these when working with patients. It is also important for clinicians to know that patients’ perceptions and behaviour are not fixed, but can change over time, with changes to their condition, with dialogue and increased understanding. Patients want this interaction with their clinicians, through “a joint effort over a period of time”.

## Background

Gout is the most common inflammatory arthropathy, affecting around 2.5 % of United Kingdom residents [1]. Its incidence and prevalence are rising, due to ageing population, increasing prevalence of metabolic syndrome, and lifestyle changes [2]. Gout is also one of the most treatable rheumatological conditions [3, 4]. Current international guidelines recommend treatment of acute attacks to reduce pain and inflammation [4–6], and encourage urate-lowering therapy (ULT) for patients with two or more acute attacks per year, tophi, renal stones, radiological damage or impaired renal function [5, 6]. ULT should be titrated according to serum uric acid (SUA) levels to achieve and maintain a target of ≤360 μmol/l [5, 6].

Given the high prevalence of gout and availability of effective treatment, most patients are managed in primary care [7]. However, the gap between clinical practice and potential treatment possibilities is large [8]. Long-term management is often suboptimal, with many patients living with raised SUA levels and recurrent attacks [9–14]. Only around 30 % of patients with gout are ever prescribed ULT [1, 9, 11]. Monitoring and up-titration of ULT to achieve a target SUA level are not performed in most patients [10, 14–16].

The literature clearly demonstrates a need for improvements in the quality of care for patients with gout, but the reasons behind current suboptimal management are less clear. Patient adherence is one issue, with only 40 % of treated patients adhering to ULT [1]. 70 % of patients have gaps in ULT – the majority within the first year of treatment [17]. Previous studies have attributed the low use of ULT to poor adherence [18], clinical factors and patients’ financial concerns [19], and adverse events including ULT induced gout attacks and/or stigma [20]. In one study only 25 % of patients recently prescribed ULT were aware it was be used long-term, and only 12 % knew that it could cause attacks in the short-term [21].

Clinician concerns about allopurinol hypersensitivity syndrome, lack of knowledge about treatment, unfamiliarity with protocols for titrating ULT, and underprescribing as a consequence of other recognised barriers such as habit, lack of motivation or time have been suggested to explain low rates of treatment with ULT [20]. Improvements in the knowledge and interest of health professionals, as well as patients, are urgently needed [1, 7, 22–24].

These quantitative studies do not, however, provide an in-depth understanding of patients’ experiences and perspectives. A small number of gout studies using qualitative methods have been carried out. One such study found that patients did not use medication prescribed by their GP for a number of reasons, including concern about side-effects, not having experienced an attack for a long period of time, and lack of understanding of the chronic nature of the condition [25]. The same study found that GPs often managed gout as an acute rather than chronic condition, did not always use guidelines, and tended to assume that patients had a good understanding of the condition. A US based study using focus groups found that patients were concerned about taking medication, including side-effects and long-term issues [26].

However, proposed reasons for ULT underprescription, undertreatment and poor adherence have not been fully explored. In this article we provide evidence for these reasons, through an exploration of patient experience and understanding of ULT treatment for gout.

## Methods

We used qualitative methods to explore gout patients’ experience and understanding of the condition and its treatment.

### The sample

We interviewed a maximum variation sample [27] of 43 people with gout from across the United Kingdom. We aimed for a diverse sample, covering experiences and demographic variables thought to be most important. The interviews were also intended to be used online as a resource (healthtalk.org), therefore a geographical spread of participants was required to provide representation of experiences in England, Scotland and Wales. A list of other categories was drawn up covering the types of experiences and demographic variables considered to be important to patients and clinicians (including current age, age at diagnosis, sex, years since diagnosis). Inclusion criteria was a self-reported diagnosis of gout being given from a healthcare practitioner. Patients were recruited through General Practitioners (GPs), rheumatology clinics, gout support groups, our expert advisory panel, online advertising and snowballing through personal contacts. Interviews were transcribed and analysed concurrently with recruitment and ongoing recruitment discussed by the research team. When 43 individuals had been interviewed, it was agreed that a point of thematic saturation had been reached and that the sample was sufficient to provide representation of experiences across all categories.

Table 1 shows the demographic characteristics of our final sample.

**Table 1:** Sociodemographic characteristics of sample

	Men (%)	Women (%)	Total (%)
	(*n* = 29)	(*n* = 14)	(*n* = 43)
Age group at interview (years):			
30–49	4 (14)	3 (21)	7 (16)
50–69	16 (56)	7 (50)	23 (54)
70–89	9 (31)	4 (28)	13 (30)
Ethnicity/Nationality:			
White	27 (93)	13 (92)	40 (92)
Asian British	2 (7)	1 (7)	3 (7)
Living arrangements:			
Living alone	2 (7)	4 (29)	6 (14)
Living with one other person	20 (69)	6 (43)	26 (60)
Living with more than one other person	7 (24)	4 (29)	11 (26)
Current work status:			
Retired	16 (55)	9 (64)	25 (58)
Full-time work	6 (21)	5 (36)	11 (26)
Part-time work	5 (17)	-	5 (12)
Student (higher education)	1 (3)	-	1 (2)
Not working for health reasons	1 (3)	-	1 (2)
Time since diagnosis (years):			
1–5	4 (14)	6 (43)	10 (23)
6–10	2 (7)	4 (29)	6 (14)
11–15	6 (21)	3 (21)	9 (21)
16 or more	17 (59)	1 (7)	18 (42)
Age group at diagnosis (years):			
< 30	1 (3)	1 (7)	2 (5)
30–49	19 (66)	4 (29)	23 (53)
50–69	9 (31)	7 (50)	16 (37)
70–89	-	2 (14)	2 (5)
Attacks in past 12 months:			
0	10 (3)	4 (29)	14 (33)
1–4	16 (55)	6 (43)	22 (51)
5–9	1 (3)	1 (7)	2 (5)
≥ 10	2 (7)	3 (21)	5 (12)

### Data collection

An experienced qualitative researcher (JL) conducted face-to-face semi-structured interviews with 43 participants in their homes or workplaces. Interviews lasted between 25 min and two hours. Using a narrative approach [28], the interviews focused on the oral history of each person’s experience of gout in order to identify their concerns, meanings and priorities. The interviewer used additional questions to prompt reflection on areas of interest identified through the literature review and/or advisory group. (Full topic guide available on request). Using this approach meant that unanticipated areas could be explored as they arose, thus identifying salient issues for people with gout.

### Data analysis

Following informed consent, interviews were audio or video recorded depending on participant preference, transcribed and checked (by JL). JL coded all transcripts under broad categories, using NVivo 10 (QSR) to assist with data management. A second researcher (SP) checked these to identify any additional codes. Analytic themes relating to specific topics, including long-term treatment – the focus of this paper – were then developed further, through discussion with main authors (JR and JL) [29]. Extracts from interviews and summaries of other topics are presented on http://healthtalk.org/peoples-experiences/bones-joints/gout/topics.

## Results

All participants talked about their views and experiences of treatment, and factors affecting their use of ULT. We discuss three anticipated themes (knowledge and understanding of gout; experiences of using ULT; information needs) and two emergent themes (resistance to medication and uncertainty about starting ULT). Themes are presented in an order which reflects a patient’s journey through the treatment ‘process’. Quotations illustrate typical responses and the range of experiences, in order to illuminate findings. All names are pseudonyms.

1. ***Knowledge and understanding of gout and its treatment***
  Patients’ perceptions and understanding of the nature of gout influenced their attitude towards medication. If they did not see it as serious or long-term they were less likely to take ULT.
  “*So I know gout’s never going to kill me, right. So I don’t want to be taking – I don’t want to be rattling around full of tablets all the time.*” (Andrew)
  Patients’ understanding of the nature of gout could change over time, and with experience and increased knowledge. Adam was explicit about needing to change the way he thought about gout in relation to taking medication:
  *“… it does take a period of adjustment to actually, you know, ‘I’m going to be doing this for the rest of my life’, rather than, ‘I’ve got something in the background which flares up occasionally.”*
  Increased knowledge of the long-term effects of gout can move a patient towards a higher likelihood of taking ULT, illustrated by Vinay and George:
  “*The doctor said if you don’t take any medication it will get worse and it can affect your joints, I mean permanent damage. So I had no choice, I have to take the medication.*” (Vinay)
  “*Don’t realise when you get it, the effect that its having on you, and I had some quite severe attacks in my hands as well as my feet, and that’s left me now with permanent damage, which I didn’t realise was going to happen.[…] The pain goes away but the effects of the attacks of the acid and crystals doesn’t go away, it remains there*.” (George)
2. ***Resistance to medication***
  Resistance to medication was a key issue and a major influence on the likelihood of starting to take ULT. Patients’ explanations and descriptions of their medication resistance include the long-term nature of treatment, sense of identity, dislike of multiple medications, and desire for self-management.
  “*If I have a headache I just put up with it [laughs] whereas I’ve got friends who will swallow any manner of things, you know, just to avoid a pain of some sort or another. It’s just the way I am.*” (Gail)
  “*Well I didn’t really want to be on medicine for the rest of my life, which is what they were suggesting.* ” (Linda)
  Some participants, although reluctant to take long-term medication, were less resistant to short-term treatment, although this form of self-management also showed aspects of general medication resistance [30, 31], for example, holding tablets ‘in reserve’. Maintaining a sense of control was important for people who wanted to self-manage: in Gail’s case, she recognised her GP’s support in this:
  “*I’ve been allowed to be in charge of my own destiny. To be fair, the doctor has probably mentioned it before last year […] he hadn’t just left me for ten years […] But I suppose because of the way I am, I am so anti taking things that probably it was a case of me coming round to accepting that I didn’t have any other options. But, as I say, it’s always easier to be wiser after the event […] maybe the medication would have been a better option a bit sooner, in my experience*”.
3. ***Uncertainty about whether and/or when to start ULT***
  Participants expressed the idea that a certain number of gout attacks was acceptable to them. The acceptable number of attacks varied between patients, as would be expected, for example, ‘*infrequent*’ (Joanne), ‘*once or twice a year*’ (Jason) to ‘*every 3 months*’ (Simon). Patients who considered a certain number of attacks acceptable were willing to self-manage by using medication for acute attacks. The perceived acceptability of a certain number of attacks may lead to patients continuing to experience regular attacks, with the view that their health professional supports this: indeed the guidelines recommend a ‘wait and see’ approach. The corollary of an ‘acceptable’ number of attacks is, of course, the unacceptable number - that is, the number of attacks that can precipitate someone into taking long-term medication, as expressed by Gail:
  “*I think I read somewhere that the current thinking on gout is that if you’ve had so many flare-ups – I don’t know what the number is – that the next step is medication.*”
  An increase in the frequency, severity, spread or impact of attacks was a key factor in moving a patient towards long-term treatment, even if they had initially resisted this route.
  “*Last year, I was getting too many instances and it was spoiling things, because I have a hobby that I enjoy and if I’d got gout I couldn’t pursue it until the gout had gone. And I actually came round to thinking that I needed to do something more about it than just trying to manage my diet and dealing with episodes when they occurred.*” (Gail)
4. ***Patients’ experiences of taking ULT***
  A key emerging theme was patients’ feeling that the process of achieving the right dosage was one of ‘trial and error’. Patients reported starting out on a low dose of allopurinol and then moving up, in conjunction with SUA level tests, until they reached optimum dosage. This is in line with current guidelines and would not be unexpected by clinicians, but for patients it could be frustrating, particularly when combined with lack of explanation, illustrated by Eric:
  “*Started on 100 mg first and it didn’t improve. And I was taking the painkilling tablet at the same time. But when I was taking the 100 mg, your mind thinks […] ‘Why am I taking?’ It gets worse. ‘Why is it getting worse?’ And it gets frustrating and you go to the doctors and he ups it to 200 and it still doesn’t go away and you’re still taking the painkiller. And he ups it again to 300 and it was still…. It just seems like it gets a balance and it’s very difficult to understand why, what’s actually going on.*”
  Other patients reported that they did not get to optimum dosage, or reached a point where uric acid levels were normal, but attacks were continuing. Some, such as Linda, were not able to get through initial attack:
  “*Every time I went on it I had a flare-up of gout, which they told me to expect, but it was so bad that I just wanted to get off it. […] They sort of explained, you know, how the chemicals work, and it’s normal to get a flare-up but it will go. But I suppose I wasn’t patient enough to deal with that.*” (Linda)
5. ***Desire for information and monitoring***
  Most participants knew little about gout before diagnosis and recalled little written information from their GP. As described in Section 1, information is a key factor in patients’ decisions to start and continue taking ULT. Patients wanted information on causes of gout, including the role (if any) of diet, relationship between urate levels and gout attacks, and the implications of taking medicine for the rest of their lives. In particular, the impact of long-term medication was sometimes seen as being underestimated by the health care practitioner, illustrated in Adam’s report of his conversation with his GP:
  “*When my attacks became more frequent, I went back to the GP to ask for a referral back to the rheumatologist to discuss, you know, ‘Is this the time to start allopurinol?’ And my GP initially refused to refer me, he said we didn’t need to - ‘I’ll just put you straight on allopurinol’. And I remember, sort of having a conversation, ‘Well, actually, you know, I’d like to discuss….’ Because I didn’t like the idea at the time of going on a medication for the rest of my life. […] It is a shift in the way of thinking about the illness that you’ve got. The prospect of ‘I’ve got to take this for the rest of my life’, is, it was very difficult to adjust to. And I said that to my GP, and he matter-of-factly said, ‘Oh, lots of people take tablets for the rest of their life’*. [Adam]
  Other patients reported similar feelings:
  “*I think when you’ve had a diagnosis, it would be really helpful to sit down quite quickly with a doctor […] to actually talk through what your options are…*” (Linda)
  “*… so I think you need that sort of an approach to try and help the person understand and get it under control. So it’s like a joint effort over a period of time, rather than just a one-off visit after you’ve identified the problem*.” (Henry)
  The desire for an ongoing dialogue with a health care professional, especially while establishing a new regime, is also reflected in patients’ desire for long-term monitoring and review of the condition and treatment.

## Discussion

**Fig. 1:**
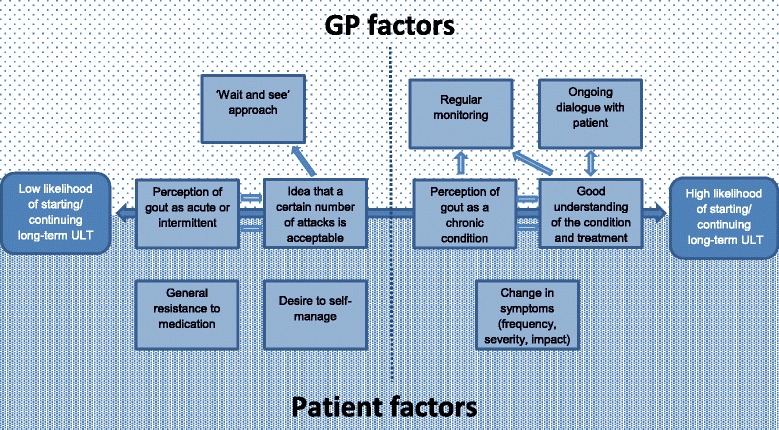
Factors affecting patients’ use of urate-lowering therapy (ULT) for long-term treatment of gout

Figure 1 presents our findings in a model, highlighting factors that influence how likely a person with gout is to take ULT. The model demonstrates the complexity of, and interaction between, factors that may explain low uptake of ULT and suggest what might help a patient to make an informed choice about medication. The model includes patient and GP factors: GP factors are the patients’ understanding and experience of interaction with their GP, which they reported influencing their beliefs or behaviour.

Although international guidelines encourage ULT for people who have more than two attacks of gout in a year, only 30 % of patients are ever prescribed it [1, 9, 11]. This study highlights the complexity of factors influencing the uptake of ULT for long-term treatment of gout. Using a qualitative approach, with in-depth interviews, enables an understanding of this complexity.

In particular, our study provides a detailed explanation of poor adherence (as reported by patients) but also explains why patients may resist being prescribed ULT in the first place. Previous literature has treated prescription as a GP issue and adherence as a patient issue: our study suggests the picture is more complex. Patient factors are involved in the initial decision to prescribe or not prescribe ULT, and GPs can influence whether patients continue to take prescribed medication.

Patient knowledge and understanding of gout influences willingness to be initially prescribed ULT. Perception of gout as intermittent, unwillingness to take medication in general and desire to self-manage through diet can all influence this. A great deal of uncertainty surrounded patients’ knowing when to start taking ULT. The ‘wait and see’ approach is in line with current guidelines. However, patients may not return to the GP after their first attack, choosing to self-manage.

Importantly, our data demonstrate that some patients moved from unwillingness to take ULT towards understanding its benefits. The key factors in this were a greater understanding of the nature of gout and/or a change in symptoms or their impact. This fits with the findings of a previous questionnaire-based study, which suggested that adherence to ULT was positively associated with greater perceived understanding of gout [32]. It also suggests that there is an important opportunity for GPs to work with patients to change their views and improve understanding. Patient education helps to achieve the therapeutic target [24] but information resources have rarely addressed patients’ need for information about ULT [19, 33]. While the idea that patients can change their view may be obvious, there are important implications for GPs. There is a need to remain open to dialogue with patients and not rule out offering ULT again even if initially rejected. In this way the initial rates of prescription may be increased.

Once ULT has been prescribed, other factors become important in determining whether patients continue to take it, with one study suggesting 75 % still taking the starting dose [11]. Our findings provide an explanation: patients sometimes stopped taking ULT when an initial attack was precipitated. Others continued to have attacks while taking ULT but did not return to their GP, while some did not have their dose adjusted, remaining on the starting dose. Patients’ experiences of taking ULT could be frustrating, when combined with lack of understanding of the process. These findings add to previous evidence that few patients understood the role of ULT [21], indicating a need for health care professionals to be more explicit in their explanations. Patients wanted information from, and dialogue with, their health care professionals, and wanted to have regular monitoring once they had chosen to take ULT to reassure them that the medication was effective and there were no adverse effects.

A limitation to this study is that the sample was predominantly white, of older age and UK based. There may be different uses of and views on ULT, and cultural reasons for this, in other populations. All the study participants self-reported a diagnosis of gout by a health care professional. The nature of the study did not allow us to verify this: previous studies have found this to be reliable [34].

Using qualitative methods enables us to answer the question of why ULT is under prescribed and has a low adherence rate, from a patient’s perspective. We used trusted methods [25–27] to explore patient experience. The sample was designed to represent diversity of experience, including those who fit the ‘typical’ gout patient profile and those who do not, rather than be statistically representative; therefore we do not present the results in frequencies, which would be misleading. The study is limited to a United Kingdom (UK) context but has the strength of being conducted by a multi-disciplinary team of social scientists and clinicians, who helped to interpret the data.

## Conclusions

Effective management of gout requires improvements in several key areas [35]. Importantly, clinicians need to ‘know how to address illness perceptions and educate their patients appropriately, so that individualized management plans can be developed on the basis of shared decision making’ [[35] p280]. Our study provides rich evidence on what these perceptions are, how they influence consequent behaviour and how clinicians can address this.

Patients’ understanding and experiences of gout and ULT are complex and it is important for clinicians to be aware of these. Patients’ perceptions and behaviour are not fixed, but can change over time, with changes to their condition, with dialogue and with increased understanding. Patients want this interaction with their clinicians, summed up by Henry’s phrase – “a joint effort over a period of time”. Clinicians can support patients’ decision-making with information about the role of ULT in long-term treatment, including expectations and processes within the treatment pathway. GPs could refer their patients to websites such as Arthritis Research UK [http://www.arthritisresearchuk.org/arthritis-information/conditions/gout.aspx] and the healthtalk website section on peoples’ experiences of gout: http://healthtalkonline.org/peoples-experiences/bones-joints/gout/topics].

Patients’ experiences, as reported in our study, not only provide insight into reasons for non-adherence, but also highlight other factors that influence if, when, and how ULT is initiated and managed, including the role that clinicians have played in this process.

## Abbreviations

GP, general practitioner; SUA, serum uric acid; ULT, urate lowering therapy

## References

[1] Kuo C-F, Grainge MJ, Mallen C, Zhang W, Doherty M (2014). Rising burden of gout in the UK but continuing suboptimal management: a nationwide population study. Ann Rheum Dis.

[2] Roddy E, Zhang W, Doherty M (2007). The changing epidemiology of gout. Nat Clin Pract Rheumatol.

[3] Zhang W, Doherty M, Pascual E, Bardin T, Barskova V, Conaghan P, EULAR Standing Committee for International Clinical Studies Including Therapeutics (2006). EULAR evidence based recommendations for gout. Part I: diagnosis. Report of a task force of the Standing Committee for International Clinical Studies Including Therapeutics (ESCISIT). Ann Rheum Dis.

[4] Jordan KM, Cameron JS, Snaith M, Zhang W, Doherty M, Seckl J, British Society for Rheumatology and British Health Professionals in Rheumatology Standards, Guidelines and Audit Working Group (SGAWG) (2007). British Society for Rheumatology and British Health Professionals in Rheumatology guideline for the management of gout. Rheumatol (Oxford).

[5] Khanna D, Khanna PP, Fitzgerald JD, Singh MK, Mae S, Neogi T (2012). 2012 American College of Rheumatology guidelines for management of gout. Part 2: therapy and antiinflammatory prophylaxis of acute gouty arthritis. Arthritis Care Res.

[6] Zhang W, Doherty M, Bardin T, Pascual E, Barskova V, Conaghan P (2006). EULAR evidence based recommendations for gout. Part II: Management. Report of a task force of the EULAR Standing Committee for International Clinical Studies Including Therapeutics (ESCISIT). Ann Rheum Dis.

[7] Rees F, Jenkins W, Doherty M (2013). Patients with gout adhere to curative treatment if informed appropriately: proof-of-concept observational study. Ann Rheum Dis.

[8] Pascual E, Sivera F (2007). Why is gout so poorly managed?. Ann Rheum Dis.

[9] Roddy E, Zhang W, Doherty M (2007). Concordance of the management of chronic gout in a UK primary-care population with the EULAR gout recommendations. Ann Rheum Dis.

[10] Annemans L, Spaepen E, Gaskin M, Bonnemarie M, Malier V, Gilbert T (2008). Gout in the UK and Germany: prevalence, comorbidities and management in general practice 2000–2005. Ann Rheum Dis.

[11] Cottrell E, Crabtree V, Edwards J, Roddy E (2013). Improvement in the management of gout is vital and overdue: an audit from a UK primary care medical practice. BMC Family Practice.

[12] Mikuls TR, Farrar JT, Bilker WB, Fernandes S, Saag KG (2005). Suboptimal physician adherence to quality indicators for the management of gout and asymptomatic hyperuricaemia: results from the UK General Practice Research Database (GPRD). Rheumatol (Oxford).

[13] Neogi T, Hunter DJ, Chaisson CE, Allensworth-Davies D, Zhang Y (2006). Frequency and predictors of inappropriate management of recurrent gout attacks in a longitudinal study. J Rheumatol.

[14] Wall GC, Koenigsfeld CF, Hegge KA, Bottenberg MM (2010). Adherence to treatment guidelines in two primary care populations with gout. Rheumatol Int.

[15] Pal B, Foxall M, Dysart T, Carey F, Whittaker M (2000). How is gout managed in primary care? A review of current practice and proposed guidelines. Clin Rheumatol.

[16] Reaves E, Arroll B (2014). Management of gout in a South Auckland general practice. J Prim Health Care.

[17] Harrold LR, Andrade SE, Briesacher B, Raebel MA, Fouayzi H, Yood RA (2010). The dynamics of chronic gout treatment: medication gaps and return to therapy. Am J Med.

[18] Reach G (2011). Treatment adherence in patients with gout. Joint Bone Spine.

[19] Harrold LR, Mazor KM, Velten S, Ockene IS, Yood R (2010). Patients and providers view gout differently: a qualitative study. Chronic Illness.

[20] Lipworth W, Kerridge I, Brett B, Day R (2011). How clinical and research failures lead to suboptimal prescribing: the example of chronic gout. BMJ.

[21] Harrold LR, Mazor KM, Peterson D, Naz N, Firneno C, Yood RA (2012). Patients’ knowledge and beliefs concerning gout and its treatment: a population based study. BMC Musculoskeletal Disorders.

[22] Doherty M, Jansen TL, Nuki G, Pascual E, Perez-Ruiz F, Punzi L (2012). Gout: why is this curable disease so seldom cured?. Ann Rheum Dis.

[23] Li Q-H, Dai L, Li Z-X, Liu H-J, Zou C-J, Ou-Yang X (2013). Questionnaire survey evaluating disease-related knowledge for 149 primary gout patients and 184 doctors in south China. Clin Rheumatol.

[24] Singh JA (2009). Quality of life and quality of care for patients with gout. Curr Rheumatol Rep.

[25] Spencer K, Carr A, Doherty M (2012). Patient and provider barriers to effective management of gout in general practice: a qualitative study. Ann Rheum Dis.

[26] Singh JA (2014). The impact of gout on patient’s lives: a study of African-American and Caucasian men and women with gout. Arthritis Res Ther.

[27] Patton M (1990). Qualitative evaluation and research methods.

[28] Mishler EG (1991). Research interviewing: context and narrative.

[29] Ziebland S, McPherson A (2006). Making sense of qualitative data analysis: an introduction with illustrations from DIPEx (personal experiences of health and illness). Medical Education.

[30] Pound P, Britten N, Morgan M, Yardley L, Pope C, Daker-White G, Campbell R (2005). Resisting medicines: a synthesis of qualitative studies of medicine taking. Soc Sci Med.

[31] Townsend A, Wyke S, Hunt K (2003). Managing multiple morbidity in mid-life: a qualitative study of attitudes to drug use. BMJ.

[32] Dalbeth N, Petrie KJ, Chong J, Leung W, Chequdi R, McQueent FM, Taylor WJ, House M (2011). lllness perceptions in patients with gout and the relationship with progression of musculoskeletal disability. Arthritis Care & Research.

[33] Robinson PC, Schumacher HR (2013). A qualitative and quantitative analysis of the characteristics of gout patient education resources. Clin Rheumatol.

[34] McAdams MA, Maynard JW, Baer AN, Köttgen A, Clipp S, Coresh J, Gelber AC (2011). Reliability and sensitivity of the self-report of physician-diagnosed gout in the campaign against cancer and heart disease and the atherosclerosis risk in the community cohorts. J Rheumatol.

[35] Rees F, Hui M, Doherty M (2014). Optimizing current treatment of gout. Nat. Rev. Rheumatol.

